# Book Review: Heise, M. *Chikungunya Virus*; Springer Nature Switzerland AG.: Cham, Switzerland, 2022; ISBN: 978-3-030-90610-8

**DOI:** 10.3390/v16101629

**Published:** 2024-10-17

**Authors:** Isak Roberth Akollo, Vernando Yanry Lameky

**Affiliations:** Department of Nursing, Universitas Kristen Indonesia Maluku, Maluku 97115, Indonesia; ukim@yahoo.com

## 1. Introduction

Chikungunya virus (CHIKV) is a type of alphavirus that is transmitted by mosquitoes. This virus was first isolated from patient serum in 1952 in Tanzania. In those worst affected, the clinical symptoms of CHIKV infection are sudden fever accompanied by joint pain. Apart from that, there are also other symptoms such as polyarthralgia, rash, arthritis, myalgia, and headaches [1].

CHIKV’s ability to spread efficiently and its high rate of symptom onset can result in the rapid development of high local case numbers that can quickly overwhelm public health systems and drive transmission of the virus into new areas. Mortality from CHIKV is generally low, but CHIKV infection causes severe polyarthralgia and myalgias. Outbreaks of arthritis caused by CHIKV can have a significant impact on quality of life and can strain local healthcare resources. In addition, arthralgia caused by the virus can persist for months to years in some infected individuals, leading to long-term negative impacts on quality of life [1].

## 2. Discussion

The uniqueness of this book lies in its coverage of advances in molecular immunology, medical microbiology, virology, and biotechnology related to CHIKV. (1) The book is written and curated by dedicated experts in their fields. (2) It presents research findings on CHIKV. (3) It combines basic knowledge with current research results and provides readers with important insights into CHIKV from historical and contemporary perspectives. (4) This book can be a first introduction to CHIKV for students and a reading resource for researchers, health professionals, and policymakers. This book consists of five chapters.

Chapter 1 of this book reviews the molecular virology of Chikungunya virus and is written by Karya I. Folov and E.I. Frolova (Department of Microbiology, University of Alabama). One of the most interesting structures of CHIKV to discuss is its nonstructural proteins. CHIKV nonstructural proteins consist of four types, namely nsP1, nsP2, nsP3, and nsP4, which have roles in the virus’s life cycle, pathogenesis, and immune response evasion. nsP1 is a multifunctional protein that is important in modulating the host’s immune response and CHIKV replication and pathogenesis. nsP1 plays a role in RNA capping because it has methyltransferase (MTase) and guanylyltransferase (GTase) activities. RNA capping is very important for RNA stability, translation, and evasion of the host’s immune response. This nonstructural protein is known to bind the replication complex (RC) to the inner surface of the plasma membrane, which can facilitate efficient viral genome replication. nsP1 also plays a role in modulating host responses by inducing plasma membrane rearrangements and interfering with host proteins that inhibit viral particle release, the mechanism of which is unclear. The new study by Law MCY and colleagues identifies the novel de-capping activity of nsP1, which allows it to remove the m7G cap from various RNA substrates, including both viral and cellular mRNAs. This activity is significant as it can lead to the generation of 5′ diphosphate RNA ends, which are known to activate the RIG-I-mediated immune response, thereby influencing host antiviral defenses [2]. nsP2 is a protein that is important in the replication and evasion of the host’s immune response. nsP2 functions as a protease that degrades the RPB1 subunit of RNA polymerase II through polyubiquitination, which allows CHKIV to manipulate the host cell’s cellular machinery to support its replication. CHIKV infection can cause RPB1 degradation, the inhibition of STAT1 phosphorylation and its translocation into the nucleus, and the inhibition of host cell transcription, resulting in proteins that play a role in host defense not being produced. nsP3 is a multifunctional protein that plays an important role in CHIKV replication and pathogenesis. nsP3 can interact with G3BP (GTPase activating protein) so that the virus manipulates the host cellular process to support its replication. In CHIKV-infected cells, nsP3 plays a role in inhibiting transcription, which causes cytoplasmic retention of various core proteins. nsP4 functions as an RNA polymerase to synthesize the viral RNA genome. This protein has two domains, namely the N-terminal domain involved in the assembly and function of the replication complex (RC) and the C-terminal domain that shows RdRp activity, which is important for RNA synthesis. nsP4 also has adenylyl-transferase activity, which is important for the stability of viral RNA and the function of the 3′ cis-acting sequence (CSE) as a promoter for negative-strand RNA synthesis.

Chapter 2 of the book is written by Guillaume Carissimo (Singapore Immunology Network, Agency for Science, Technology, and Research, Biopolis, Singapore, Singapore) and Lisa FP Ng (Institute of Infection and Global Health, University of Liverpool, Liverpool, UK), who discuss the current understanding of the molecular pathogenesis using the tools of Chikungunya virus research. One of the interesting tools in studying the immunopathogenesis of CHIKV is the use of mouse models. The use of mouse models has yielded promising results, although they do not fully reflect the clinical symptoms in humans. The C56BL/6 mouse model of arthritis is the model that most closely resembles human inflammatory arthritis. Studies in mouse models have revealed the importance of innate immunity in CHIKV infection. Despite having high viral loads, mice lacking adaptive immunity (B and T cells) are not susceptible to CHIKV infection. Conversely, mice lacking a functional type I IFN pathway are highly susceptible to CHIKV infection. Certain genes, such as interferon-stimulated genes (ISGs) (e.g., ISG15, Tetherin, or Viperin), have been identified as having a protective role against CHIKV, according to studies using mouse models.

Chapter 3 of the book is written by Mary K. McCarthy, Bennett JJ Davenport, and Thomas E. Morrison (Department of Immunology and Microbiology, University of Colorado School of Medicine), who discuss some of the findings of chronic Chikungunya virus disease, particularly the immune response that leads to chronic clinical symptoms. The hallmarks of chronic disease are arthralgia and arthritis. The development of chronic clinical symptoms is influenced by T cells and viral load. The dysregulation of CD4 regulatory T cells (Tregs) contributes to chronic CHIKV disease. Proinflammatory cytokines are produced in excess during CHIKV infection, which affects Treg cell function and reduces its ability to suppress effector T cell activity. Excess effector T lymphocytes and proinflammatory cytokines exacerbate chronic arthritis and other clinical symptoms. According to recent research by Brito RM de M and colleagues, CHIKV infection can cause an increase in IL-10 production and a decrease in TGF-β levels, which are important for Treg function. A reduction in TGF-β is indicative of impaired Treg differentiation, which may lead to uncontrolled viral replication and inflammation [3]. An excessive viral load during the acute phase of infection is thought to trigger a persistent immune response and joint inflammation. In some cases of CHIKV infection, an autoimmune reaction occurs, as the immune system inappropriately targets the body’s joint tissue, leading to joint damage and long-term complications such as cartilage degradation or synovitis, which can lead to chronic pain such as arthritis and chronic arthralgia.

Chapter 4 of this book reviews some of the new aspects of CHIKV vaccine development and is written by Victor R. DeFilippis (Vaccine and Gene Therapy Institute, Oregon Health and Science University). One of the newest and most exciting aspects of CHIKV vaccine development is the use of chimeric viral vectors. These vectors are designed to deliver CHIKV antigens without posing a risk to mammalian cells because they contain regions of the CHIKV genome that encode antigenic proteins which enter cells in vivo but do not produce progeny or undergo only one round of replication, thus reducing the risk of causing disease. These viral platforms can trigger both cellular and humoral immune responses; by exploiting cellular protein synthesis and processing pathways, they lead to antigen presentation via MHC class I and subsequent T cell activation. These viral particles have characteristics in common with adjuvants, which means they have the ability to mount an immune response. This technology is a new concept that is expected to improve the efficacy of CHIKV vaccines. In addition, this chapter also describes several vaccine technologies used, such as live attenuated vaccines, virus-like particles (VLPs), nucleic acid-based vaccines, and inert antigen vaccines.

Chapter 5 of this book describes smallmolecule inhibitors targeting Chikungunya virus and is written by Nicole Haese, Jhon Powers, and Daniel N. Streblow DeFilippis (Vaccine and Gene Therapy Institute, Oregon Health and Science University). Several inhibitors have been developed to target different stages of the CHIKV life cycle, including virus entry, replication, and virus assembly. Some of the most interesting inhibitors discussed in this chapter are auranofin and ganetespib. Auranofin inhibits protein disulfide isomerase (PDI), which plays a role in facilitating viral protein folding, virus maturation, and cell fusion with the virus. Auranofin has shown promising results in preclinical models, demonstrating a therapeutic index indicating efficacy against CHIKV. Ganetespib targets Hsp-90, which plays a role in stabilizing viral proteins, including nonstructural proteins (nsPs) that are essential for CHIKV replication. The inhibition of Hsp-90 will inhibit CHIKV replication and reduce the amount of virus in the host. However, there are several challenges in the development of inhibitors. For example, the virus mutates rapidly, thus quickly developing resistance to most single drugs; combination therapy is therefore required to enhance efficacy and reduce the likelihood of resistance. Auranofin and ganetespib are two of the inhibitors whose potential for use in combination therapy has been discussed in this chapter. Nevertheless, the precise inhibitors that can be mixed have not been fully clarified. A recent study by Davuluri KS and colleagues has identified 2-fluoro adenine (2-FA), emetine (EM), lomibuvir (L), enalaprilat (EN), metyrapone (M), and resveratrol (R) as potential CHIKV inhibitors. According to the results, when compared to single medications, combinations like 2-FA plus emetine, metyrapone, or enalaprilat exhibit notable inhibitory activity against CHIKV [4].

This book offers several advantages. (1) It explores various aspects of Chikungunya virus, such as the genome, nonstructural proteins, replication complexes, interactions with host factors, and mechanisms involved in RNA synthesis and viral replication. (2) It provides information on the use of research tools such as in vitro studies, animal models, genomic screening, and studying the interactions between viruses, host factors, and vectors. (3) It provides an overview of Chikungunya virus, focusing on various aspects such as epidemiology, pathogenesis, clinical manifestations, immune responses, and potential treatment strategies. (4) It explores various vaccine platforms, progress, and challenges in vaccine development against Chikungunya virus (CHIKV). (5) It investigates small-molecule inhibitors, such as those that block nsP3, viral entry, and viral capsid formation, as viable therapeutic approaches against CHIKV infection.

This book also has several weaknesses. (1) As research in virology and immunology advances rapidly, the content of this book may quickly become outdated. Discoveries, technologies, or treatment strategies may have emerged since this book was published, limiting its relevance to current developments in the field. An example of such discoveries include RA-0002034 and oxibendazole (OBZ) inhibitors that target nsP2 protease [5,6]. (2) This book may contain technical terms or complex scientific concepts that may be difficult to understand for readers who do not have a background in microbiology, immunology, or related fields. This may hinder the accessibility of this book to a wider audience interested in studying Chikungunya virus.
